# Spatial frequency–based correction of the spherical aberration in living brain imaging

**DOI:** 10.1093/jmicro/dfad035

**Published:** 2023-06-14

**Authors:** Aoi Gohma, Naoya Adachi, Yasuo Yonemaru, Daiki Horiba, Kaori Higuchi, Daisuke Nishiwaki, Eiji Yokoi, Yoshihiro Ue, Atsushi Miyawaki, Hiromu Monai

**Affiliations:** Department of Biological Sciences, Graduate School of Humanities and Sciences, Ochanomizu University, Ohtsuka, Bunkyo-ku, Tokyo 112-8610, Japan; RIKEN Center for Brain Science-Evident Open Collaboration Center, Center for Brain Science (CBS), RIKEN, 2-1, Hirosawa, Wako-shi, Saitama 351-0106, Japan; RIKEN Center for Brain Science-Evident Open Collaboration Center, Center for Brain Science (CBS), RIKEN, 2-1, Hirosawa, Wako-shi, Saitama 351-0106, Japan; RIKEN Center for Brain Science-Evident Open Collaboration Center, Center for Brain Science (CBS), RIKEN, 2-1, Hirosawa, Wako-shi, Saitama 351-0106, Japan; RIKEN Center for Brain Science-Evident Open Collaboration Center, Center for Brain Science (CBS), RIKEN, 2-1, Hirosawa, Wako-shi, Saitama 351-0106, Japan; RIKEN Center for Brain Science-Evident Open Collaboration Center, Center for Brain Science (CBS), RIKEN, 2-1, Hirosawa, Wako-shi, Saitama 351-0106, Japan; RIKEN Center for Brain Science-Evident Open Collaboration Center, Center for Brain Science (CBS), RIKEN, 2-1, Hirosawa, Wako-shi, Saitama 351-0106, Japan; RIKEN Center for Brain Science-Evident Open Collaboration Center, Center for Brain Science (CBS), RIKEN, 2-1, Hirosawa, Wako-shi, Saitama 351-0106, Japan; RIKEN Center for Brain Science-Evident Open Collaboration Center, Center for Brain Science (CBS), RIKEN, 2-1, Hirosawa, Wako-shi, Saitama 351-0106, Japan; RIKEN Center for Brain Science-Evident Open Collaboration Center, Center for Brain Science (CBS), RIKEN, 2-1, Hirosawa, Wako-shi, Saitama 351-0106, Japan; Department of Biological Sciences, Graduate School of Humanities and Sciences, Ochanomizu University, Ohtsuka, Bunkyo-ku, Tokyo 112-8610, Japan; RIKEN Center for Brain Science-Evident Open Collaboration Center, Center for Brain Science (CBS), RIKEN, 2-1, Hirosawa, Wako-shi, Saitama 351-0106, Japan

**Keywords:** spherical aberration, correction collar, objective lens, refractive index

## Abstract

Optical errors, including spherical aberrations, hinder high-resolution imaging of biological samples due to biochemical components and physical properties. We developed the Deep-C microscope system to achieve aberration-free images, employing a motorized correction collar and contrast-based calculations. However, current contrast-maximization techniques, such as the Brenner gradient method, inadequately assess specific frequency bands. The Peak-C method addresses this issue, but its arbitrary neighbor selection and susceptibility to the noise limit its effectiveness. In this paper, we emphasize the importance of a broad spatial frequency range for accurate spherical aberration correction and propose Peak-F. This spatial frequency–based system utilizes a fast Fourier transform as a bandpass filter. This approach overcomes Peak-C’s limitations and comprehensively covers the low-frequency domain of image spatial frequencies.

## Introduction

Optical errors often hamper high-resolution optical imaging of biological samples. Such errors are due to biochemical components such as water, proteins and lipids as well as physical properties such as anisotropy and the biological tissue’s refractive index (RI). Additionally, induced tissue scattering limits image acquisition in deeper regions or aged tissue. Among the optical errors, spherical aberrations are due to mismatched RIs between the immersion medium (e.g. water) and the sample, causing photons to converge into two focal planes [1–5]. A correction collar, usually attached to the objective lens with a high numerical aperture, is a revolving optical mechanism to reduce the mismatch between RIs [6,7]. It adjusts the distance of the inner lens to correct spherical aberrations. In the case of a histological sample, for example, the correction collar is adjusted to ‘θ = 0 degrees’, indicating that 0.17 μm corresponds to the thickness of the cover glass. However, changing the correction collar is difficult for thick samples because there is no objective measurement of the necessary rotation to eliminate spherical aberrations. To obtain spherical aberration–free images, we developed a fully automated spherical aberration compensation microscope system called Deep-C [8]. This system has a motorized correction collar, and the contrast of the entire image quantifies spherical aberrations at a given rotation angle.

A contrast-based calculation is often used to determine the sharpness of an image to maximize its contrast value since a clear image has a high contrast value, whereas a blurred one has a low contrast value. For example, out-of-focus (no focus), an optical phenomenon, occurs when multiple subjects are at different distances from the lens, and the light passing through the lens forms images at various locations. Having all images in focus on the same focal plane is impossible. Hence, unfocused light sources produce unclear images for the observer. Autofocusing involves adjusting the distance between the lens and the specimen to focus the light automatically [9]. To determine the optimal focal length, the Brenner gradient method is widely used to maximize the contrast [10]. In this method, the total contrast is equal to the square of the difference in intensity between neighboring pixels, which is given as follows:


(1) $${F_{{\mathrm{Brenner}}}} = {\mathrm{\,}}\mathop \sum \limits_{xy} {\left\{ {f\left( {x,y} \right) - f\left( {x + 2,y} \right)} \right\}^2}$$

Although spherical aberrations and defocusing stem from distinct physical phenomena, both share the common characteristic that light does not converge on a single focal point. Thus, spherical aberrations may be rectified by employing a technique to mitigate blurriness in defocused imagery, as both methodologies are predicated on maximizing image contrast intensity. Regrettably, specific frequency bands elude evaluation by merely adapting the Brenner gradient to micrographic images, attributable to the incongruity between the contrast appraisal region and the spatial frequency of the image. Ue et al. addressed this issue by computing the contrast value as the aggregate of squared disparities among 2, 3, 5, 10 and 20 neighbors [8].


(2) $${F_{{\mathrm{Peak - C}}}} = {\mathrm{\,}}\mathop \sum \limits_n \mathop \sum \limits_{xy} {\left\{ {f\left( {x,y} \right) - f\left( {x + n,y} \right)} \right\}^2}$$

where *n* = 2, 3, 5, 10 and 20.

This approach, dubbed Peak-C, accommodates an extensive spectrum of spatial frequencies. However, the selection of 2, 3, 5, 10 and 20 neighbors appears somewhat arbitrary and insufficiently comprehensive to encompass all spatial frequencies.

In this paper, we assumed that considering a wide range of spatial frequencies is necessary for accurate spherical aberration correction. We investigate the effects of spatial frequency, particularly in the low-frequency domain, and highlight potential issues associated with using Peak-C, such as susceptibility to noise and saturation. Additionally, the inclusion of the all-frequency domain into the Peak-C calculation will incur significant computational resources. To address these disadvantages of Peak-C, we propose Peak-F, a spatial frequency–based system designed as a bandpass filter of the spatial frequencies. By applying a fast Fourier transform (FFT) to the images, Peak-F comprehensively covers the low-frequency domain of the image spatial frequencies.

## Methods

All experimental protocols were approved by the Institutional Animal Care and Use Committee of Ochanomizu University, Japan (animal study protocols 22017). All animal experiments were performed according to the guidelines for animal experimentation of Ochanomizu University that conform with the Fundamental Guidelines for Proper Conduct of Animal Experiment and Related Activities in Academic Research Institutions (Ministry of Education, Culture, Sports, Science and Technology, Japan). Efforts were taken to minimize the number of animals used. This study was carried out in compliance with the ARRIVE guidelines.

### Surgical procedure

Male and female Thy1-YFP-H(YFP-H) transgenic mice [11] were used. Young mice were 9, 11, 25 and 25 weeks of age, while old mice were 52, 81, 83 and 91 weeks of age. Mice were housed under 12 h:12 h light:dark cycles and raised in groups of up to five. Mice were anesthetized with isoflurane (2%), and their body temperature was maintained at 37°C with a heating pad (BWT-100 A, Bio Research Center or TR-200, Fine Science Tools) during surgery and recording. After skull exposure, a metal frame was attached to the skull using dental acrylic (Fuji LUTE BC, GC, Tokyo, Japan; Super Bond C&B, Sunmedical, Shiga, Japan). For two-photon imaging, a craniotomy (2.7 mm diameter) was made above the visual cortex (Anterior-Posterior axis −2.0 mm, Medial-Lateral axis +2.5 mm). Then, the dura mater was surgically removed. Next, the craniotomy was gently sealed with a thin glass coverslip (2.7 × 2.7 mm, thickness: 0.12–0.17 mm, Matsunami Glass, Osaka, Japan). Finally, the cranial window was secured with dental cement (Fuji LUTE BC, GC, Tokyo, Japan; Super Bond C&B, Sunmedical, Shiga, Japan).

### Two-photon excitation imaging

**Fig. 1.**
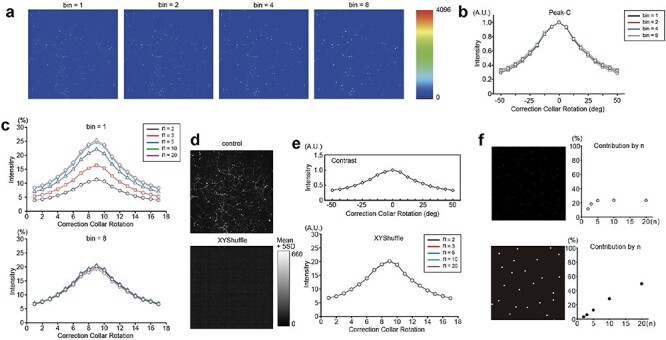
The determination of the optimal correction collar position with the Peak-C algorithm based on the image contrast. (a) Binning was performed on the benchmark image; 256 × 256 (bin = 2), 128 × 128 (bin = 4) and 64 × 64 pixel (bin = 8) images were created. (b) Intensities for each of the 17 correction collar positions calculated with the Peak-C algorithm for the images in a. The vertical axis is the contrast intensity, the horizontal axis is the rotation angle of the correction collar and the intensity is normalized by the maximum value. (c) The contribution of the Peak-C algorithm at each *n* when bin = 1 and bin = 8. *n* = 2 is black, *n* = 3 is red, *n* = 5 is blue, *n* = 10 is green and *n* = 20 is purple. The vertical axis is the intensity of contrast or power, the horizontal axis is the correction collar rotation angle and the intensity is normalized by the maximum value. (d) XY shuffled image. For ease of viewing, the threshold is set to the average of the actual brightness + 5 SD, and all pixel brightness above the threshold is set to 660. (e) The intensity of each of the 17 correction collar positions was calculated with the Peak-C algorithms on the image in c. The vertical axis is the contrast intensity, the horizontal axis is the rotation angle of the correction collar, and the intensity is normalized by the maximum value. (f) The proportion of the contribution with each *n* (*n* = 2, 3, 5, 10 and 20) at the optimum correction collar position in the artificially generated image with 1.5-μm-diameter sparse bright spots (upper) and 10-μm-diameter bright spots (bottom).

Multi-photon laser scanning microscope (FVMPE-RS, Evident) equipped with an InSight laser system (Spectra-Physics, 960 nm wavelength) and an Olympus objective (FV30-AC25W, numerical aperture: 1.05, working distance: 2 mm, and immersion medium: water) was used. In Figs. 1 and 3–5, the image size is 1024 × 1024 pixels (16-bit resolution), while the image size in Fig. 6 is 1024 × 1024 pixels (8-bit resolution).

### Benchmark images

The benchmark images featured in Figs. 1 and 3–5 were previously obtained from reference [8]. In this study, we used a distinct dataset, with a focal depth of 400 µm from the brain surface, as opposed to the 700 µm depth in the earlier data, with the authors’ permission. In addition, due to variations in the optimal collection collar position across different focal planes, the optimal position in this paper differs from that in the previous results.

## Data analysis

The acquired Fluoroview .oir file was converted into a 1024 × 1024 pixel, 8-bit grayscale bitmap image. Then, the calculations were performed using MATLAB.

### Peak-C

The Peak-C algorithm was calculated using the formula from the previous study [8].

### Peak-F 1D FFT

Fast Fourier transforms (FFT) is applied on an image one column at a time from the top as a matrix. Then, it determines the optimal angle of the correction collar by comparing the total intensity of the extracted power spectrum from the frequencies ranging from 2 to 40 Hz for each correction collar position.

### Peak-F 2D FFT

2D FFT is applied the entire image. Then, it determines the optimal angle of the correction collar by comparing the total intensity of the extracted power spectrum from frequencies ranging from 2 to 40 Hz for each correction collar position.

### Adding white noise

A 1024 × 1024 random matrix was generated using the MATLAB function rand. Then, multiplying the random matrix by σ created noise images for several intensities. The noise was reproduced by adding the benchmark image, and the noise image was created. σ was set to 256, 512, 1024 or 2048. Additionally, σ was set to 0 or as a control.

### Binning

Bicubic interpolation was performed on the benchmark image. The matrix size was reshaped to 1024 × 1024 for bin = 1, 512 × 512 for bin = 2, 128 × 128 for bin = 4 and 64 × 64 for bin = 8.

### Random shuffle

The rows and columns of each pixel in the benchmark image were randomly placed using the function randperm.

### Adding saturation

The mean value + the standard deviation (SD) of the luminance of the benchmark image was used as the threshold value. Then, 500, 700, 900 or 1100 were added to the values of pixels above the threshold value. Afterwards, pixel values >4096 were set to 4096.

## Implementation to a two-photon microscope

Peak-F calculations for two-photon excitation imaging were performed by modifying the FV31S-SW software of the FVMPE-RS TruResolution (Evident) system. Especially the contrast calculation processing part of Peak-C was replaced with Peak-F. The image frequency was calculated by replacing only the contrast calculation part of Peak-C with the image frequency calculation process of Peak-F. Image frequencies were calculated using the well-known Cooley–Tukey-type FFT method.

## Results

### It is necessary to consider a wide range of spatial frequencies to perform accurate spherical aberration correction from biological images

To investigate the optimal correction collar position for spherical aberration correction, we applied binning to the benchmark image (see Methods) to change its spatial frequency while maintaining the overall contrast (Fig. 1a). We captured 17 images at different correction collar positions and calculated the contrast intensity of each image using the Peak-C algorithm (see Methods). As a result, the peak position was accurately detected in all binned images regardless of the bin size (Fig. 1b).

Next, we assessed the influence of spatial frequency on contrast intensity across various spatial frequencies. For example, in the Peak-C, we used five discrete conditions as presented by *n* values (where *n* = 2, 3, 5, 10 and 20). Unless otherwise noted, we summed up the contrast under all *n* = 2, 3, 5, 10 and 20. But we used particular *n* values in Fig. 1c, e, and f.

As shown in Fig. 1c, significant contributions were observed in low-frequency domains in the original image, particularly for *n* = 5, 10 and 20. However, at bin = 8, contributions remained consistent for all spatial frequencies. Notably, *n* = 2 with bin = 8 equals *n* = 16 with bin = 1. This consistency in *n* contributions at bin = 8 implies that the minimum *n* value, *n* = 2, corresponds to an increased length compared to bin = 1, allowing for improved capture of image structural features. As a result, spatial structural information exceeding *n* = 16 is crucial for precisely determining the optimal correction collar position for this benchmark image.

Furthermore, we demonstrated the importance of spatial structures in biological systems by randomly shuffling the pixels of the image, which resulted in no difference in the contribution of *n* (Fig. 1d–e). To clarify this possibility further, we calculated the contribution of spatial frequency in a dot pattern generated artificially (Fig. 1f). The results showed that the contribution in the low-frequency region flattened out when small structures with a diameter of 1.5 μm were present. In contrast, the contribution increased in the low-frequency region for larger structures with a diameter of ∼10 μm.

Therefore, to perform accurate spherical aberration correction in magnified optical systems, it is necessary to pay particular attention to the low spatial frequency domain of the image.

### The Peak-F algorithm, which comprehensively takes into account the low-frequency domain

In Peak-C, it is infeasible to perform calculations considering all spatial frequencies due to the high computational cost. Consequently, we devised an algorithm, Peak-F, which allows for the comprehensive analysis accounting for the spatial frequencies in images by applying a bandpass filtering process. This process involves performing a Fourier transform on the image and only considering the power in the low-frequency domain.

**Fig. 2.**
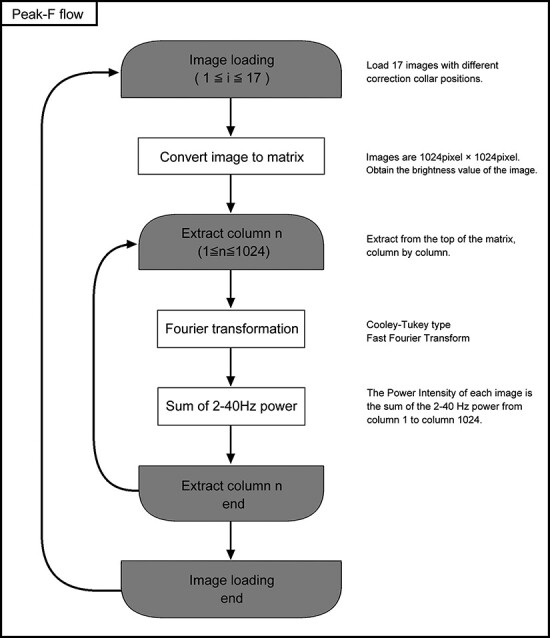
The flowchart of the Peak-F algorithm. Colored half-ellipses represent looping between the open and closed counterparts, with the number of loops denoted by ‘*i*’ and ‘*n*’. White rectangles indicate image processing and mathematical operations, with further details provided in the adjacent right column.

Peak-F was devised to aggregate the power spectrum in the 2–40 Hz range for each X-axis coordinate in the image. For instance, when an image (500 µm square) is captured with a resolution of 1024 pixels (X-axis) by 1024 pixels (Y-axis), the power spectrum of the first row along the X-axis (from the 1st to the 1024th pixel) is computed and extracted within the 2–40 Hz (12.5–512 µm) range. This extraction procedure is successively executed up to the 1024th pixel of the Y-axis and subsequently combined. The flowchart visualizes this sequential process, as shown in Fig. 2.

**Fig. 3.**
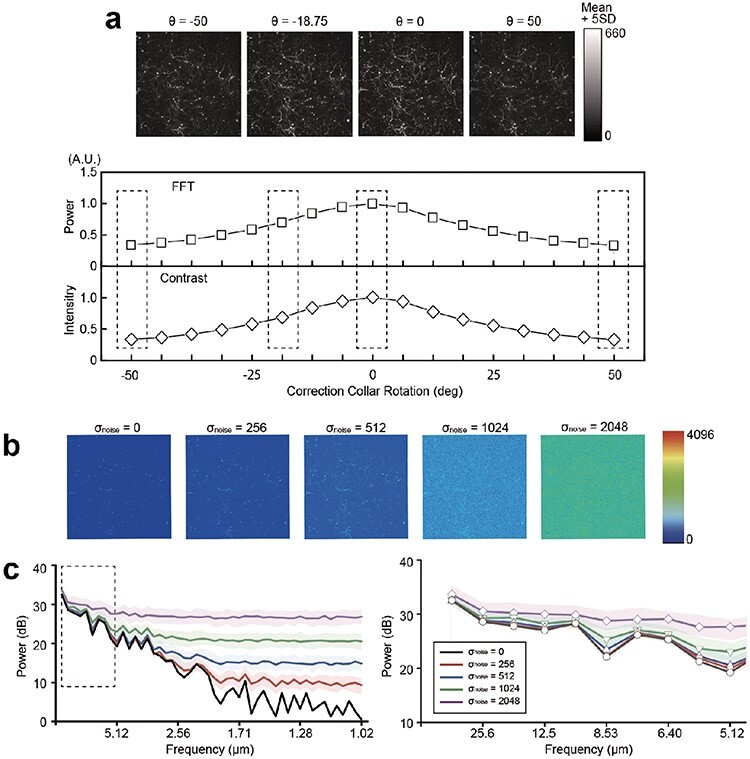
The determination of the optimal correction collar position with Peak-F algorithm based on the image spatial frequency. (a) The variation of power and contrast intensity with the angle of the correction collar. The vertical axis is the power or contrast intensity, and the horizontal axis is the rotation angle of the correction collar. (b) Benchmark image (σ = 0) and that with noise added (σ = 256, 512, 1024 and 2048). (c) Power spectrum for σ = 0 (black), 256 (red), 512 (blue), 1024 (green) and 2048 (purple). The power spectrum for the frequency range 1–40 Hz is shown in the dashed square (right). The vertical axis is power, the horizontal axis is frequency and the colored area is the standard error.

We prepared benchmark images captured from living mouse brains to evaluate whether the proposed Peak-F algorithm can accurately determine the optimal correction collar position, akin to the Peak-C algorithm. Seventeen benchmark images were acquired using a Deep-C objective with a motorized correction collar, rotating from −50° to 50° at 6.25° intervals. The images were captured at 400 µm from the cerebral cortex surface in Thy1-YFP-H-line transgenic mice. The results of Peak-F, plotting the total power in the 1–40 Hz range, revealed that the maximum power was achieved when the correction collar rotation angle was 0° (θ = 0). After applying the Peak-C algorithm to the benchmark images, it was determined that θ = 0 was the optimal correction collar position (Fig. 3a). The optimal position (θ = 0) differs from the previous findings [8] because of variations in the optimal collection collar position across different focal planes. In this study, we used a separate dataset for benchmark images with a focal depth of 400 µm from the brain surface, in contrast to the 700 µm depth in the earlier data.

Figure 3b and c elucidates the rationale for selecting a 2–40 Hz spectrum (12.5–512 µm). First, noise in deep brain tissue cannot be overlooked. Therefore, our objective was to comprehend how noise impacts the power spectrum of spatial frequencies derived from typical *in vivo* brain images captured using a two-photon microscope. We then incorporated white noise with varying intensities offline into the benchmark image (Fig. 3b). As the noise intensity (σ_noise) increased, the influence of noise became more pronounced, particularly in the high-frequency region, causing a greater deviation from the power spectrum obtained from the benchmark image. In contrast, the low-frequency component >12.5 µm (40 Hz) was noise resistant (Fig. 3c).

### Peak-F algorithm is robust to noise and saturations

**Fig. 4.**
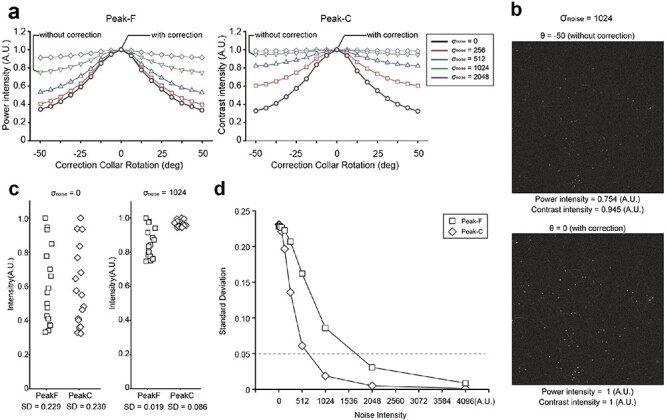
The comparison of Peak-F and Peak-C algorithms’ performance on noise-added images. (a) Intensities for each of the 17 correction collar positions calculated with the Peak-F and Peak-C algorithms for the image in Fig. 3a. The vertical axis is the power or contrast intensity, the horizontal axis is the rotation angle of the correction collar and the intensity is normalized by the maximum value. (b) The comparison of the raw image before and after correction of the spherical aberration. (c) The ability to detect peaks with no noise (σ_noise = 0) and strong noise (σ_noise = 1024) was evaluated by the variation (standard deviation) of values at 16 locations. (d) Trends in the value of standard deviation for each noise intensity.

The impact of noise becomes more noticeable when the image’s spatial frequency is <12.5 µm (Fig. 3c). Since Peak-F is designed to disregard structures <12.5 µm, we assumed it to consistently identify the optimal correction collar position, even in images with added noise. To demonstrate this, we compared the abilities of Peak-F and Peak-C in determining the optimal correction collar position while varying the noise intensity (σ) using the benchmark image from Fig. 3a, which includes artificially added white noise (Fig. 3b). We evaluated the variations in contrast or power displacement for the images captured at each correction collar position at noise intensities of σ = 0 and σ = 1024. Consequently, the difference in intensity required to detect the optimal correction collar position decreased as noise intensity increased for both algorithms. However, the difference for Peak-C was considerably smaller than that for Peak-F (Fig. 4b, SD = 0.086 vs. 0.018). In this context, we have defined an arbitrary threshold of 0.05 for the SD value, and we considered if the value falls below this threshold, it indicates that the system could not identify the optimal position. Figure 4c depicts the calculated SD for each noise intensity level, further highlighting Peak-F’s robustness to noise compared to the more vulnerable Peak-C algorithm.

**Fig. 5.**
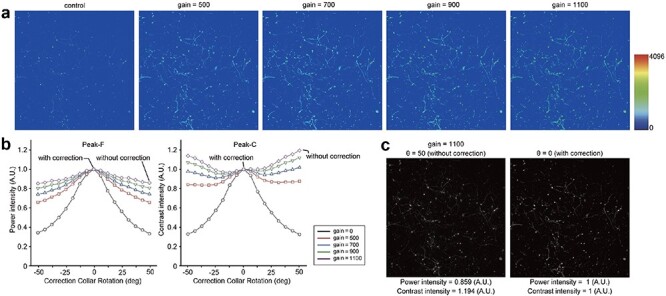
The comparison of Peak-F and Peak-C performance on saturation-added images. (a) Benchmark image (control) and that with four levels of saturation (gain = 500, 700, 900 and 1100). (b) The intensity of each of the 17 correction collar positions calculated by the Peak-F and Peak-C algorithms when saturation was applied to the benchmark image in a. The vertical axis is the power or contrast intensity, the horizontal axis is the rotation angle of the correction collar and the intensity is normalized by the maximum value of the control image. (c) The comparison of the raw image before and after correction of the spherical aberration.

Next, to demonstrate that the algorithm does not simply reflect the image’s brightness to determine the optimal correction collar position, we added saturated pixels to the benchmark image by increasing the pixel gain beyond the threshold without altering its spatial frequency (Fig. 5a). As the number of saturated pixels increased, the incorrect position was identified as the optimum using the Peak-C algorithm. In contrast, the Peak-F algorithm accurately found the optimum correction collar position even when 0.03% of pixels were saturated (Fig. 5b). This result suggests that Peak-F does not simply look at brightness but also recognizes biological structures in the image.

### Peak-F reveals optical characteristics of the brain tissue from noisy images

**Fig. 6.**
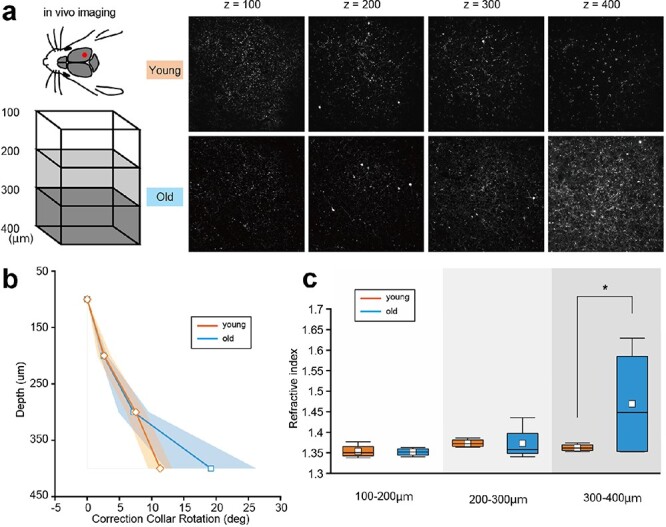
Peak-F reveals optical characteristics of the brain tissue from noisy images. (a) Images of 100, 200, 300 and 400 μm below the surface of the brain of young and old mice. (b) The mean (solid line) and standard deviation (colored area) of the optimal angle of the correction collar for young and old mice. (c) The tissue refractive index was calculated from the optimal angle of the correction collar for young and old mice. 300–400 μm showed a significant difference between young and old mice. Tests were performed with two-way analysis of variance. *P < 0.05.

The Peak-F algorithm exhibits robustness against noise and saturation in the benchmark images (Figs. 4 and 5). To demonstrate these advantages in the live images, we incorporated Peak-F into a two-photon microscope system to assess its applicability in actual *in vivo* imaging. We examined samples in both young (9, 11, 25 and 25 weeks) and old (52, 81, 83 and 93 weeks) mice. Figure 6a illustrates representative examples of a young mouse (25 weeks old) and an old mouse (93 weeks old). The images display these specimens at varying depths: 100, 200, 300 and 400 µm beneath the surface of the brain (Fig. 6a). These samples are different from the benchmark images used in Figs. 1 and 3–5. As we expected, the deeper regions in older mice appeared noisy, which might reflect the optical characteristics of the tissue, such as RIs. Ue et al. reported that our system could quantify RIs in any sample using the relationship between imaging depth and the correction collar angle [8]. Thus, by applying the Peak-F algorithm to detect the optimal correction collar angle in the noisy image, we could calculate RIs in the deeper regions of aged mice. Figure 6b illustrates the relationship between depth and the optimal correction collar angle. Converting this relationship into RIs, we identified a significant difference between young and old mice at depths of 300–400 µm (Fig. 6c, 1.36 ± 3.88E-3 vs. 1.46 ± 5.99E-2, *P* = 0.04).

## Discussion

Peak-F is a spatial frequency bandpass filter based on an FFT that extracts low-frequency boundaries from the images (Fig. 2 and 3). This approach is based on the observation that noise appears in the high-frequency region. Comparing Peak-C and Peak-F reveals that Peak-F possesses a noise-resilient nature (Fig. 4) and is stable to other disturbances such as saturation (Fig. 5). Furthermore, we incorporated Peak-F into a two-photon microscope, allowing the deep area of an aged mouse brain to be imaged (Fig. 6).

Ue et al. previously applied Peak-C to determine the optimal correction collar position from images [8]. The Peak-C algorithm calculates the optimal correction collar position based on image contrast. A contrast-based optimum search algorithm is also employed in a digital camera. Digital cameras employ reduction optics, resulting in important information in neighboring pixels [11]. Autofocusing techniques used in digital cameras involve simple subtraction between adjacent pixels, which is adequate for calculating image contrast [9]. This method is known as Brenner’s equation (Eq. 1). Conversely, microscopy uses magnifying optics, making it susceptible to high-frequency components as the difference between neighbors is minimal, and Brenner’s equation cannot accurately calculate contrast.

Therefore, Peak-C compensates for the effect of spatial frequency in contrast calculation by subtracting from multiple distant pixels (*N* = 2, 3, 5, 10 and 20). However, the impact of spatial frequency on analysis accuracy remained unclear, prompting us to analyze the contribution of each *N* (Fig. 1). The benchmark image provided uniform contributions across all frequency components at the optimal correction collar position, suggesting that Peak-C tends to calculate signals and high-frequency components, such as noise, equally. Consequently, Peak-C is vulnerable to noise. In the present study, we utilized white noise; however, it might be necessary to consider the effects of fluorescence noise and amplification noise. On the other hand, amplification noise, in the case of photomultiplier tubes, can be considered negligible due to its relatively small magnitude [12].

In contrast, the contribution of low-frequency components may depend on the object’s size. For instance, the signal in the benchmark image contained sparse dendrites with diameters of ∼1.5 μm. In an artificially generated image with 1.5 μm diameter sparse bright spots, the contribution of the low-frequency component saturated at ∼20%. However, the contribution of the low-frequency component was much larger for 10-μm-diameter bright spots resembling somata. The high-frequency domain was negligible (Fig. 1c). Thus, using only the low-frequency component for contrast calculations is sufficient for large structures such as cell bodies. However, since living organisms possess a wide variety of large structures, calculating the contrast in every frequency band is necessary to determine the optimal contrast value, which requires unrealistically high computational costs.

Whereas biological structures are concentrated in the low-frequency band, and excluding high-frequency components allows for easy separation of noise and frequency bands, the Fourier transform analysis in Peak-F is well suited for determining the optimal correction collar position. Furthermore, Peak-F is flexible and can accommodate any structure, including noise, while maintaining low computation costs. As a result, Peak-F enhances the ability to make optimal image decisions for noise and saturation with faster computational speed than Peak-C.

In the methodology presented in this paper, the change in spherical aberration is considered equivalent to the difference in the RI, assuming that the interface is flat. If the interface is not flat (i.e. samples exhibiting coma aberration), replacing the change in spherical aberration with the difference in the RI may not be feasible. However, when plotting graphs while changing the correction collar using Peak-C and Peak-F, it is believed that estimating the RI is possible, albeit with varying degrees of accuracy, if a peak occurs (the graph would become more obtuse as other aberrations increase). Nevertheless, there are no available data on the extent to which other aberrations should be considered about the correction amount applied to the objective lens used in this study. Moreover, it cannot be assumed that other aberrations’ influence is smaller than a spherical aberration. If other aberrations become more significant, estimating the RI may become challenging.

The techniques used in adaptive optics have been proposed to correct the optical error not only spherical aberrations [2,13–20] but also other more complex aberrations that will appear with tilted or curved samples, such as coma aberrations [18,20,21]. However, the optical path fluctuations must be calculated in advance to apply these adaptive optics to *in vivo* brain imaging since the brain tissue’s RIs are unknown. For example, embedded fluorescent beads with a known diameter and luminance at the given depth of the brain tissue are necessary to calculate the optical path fluctuations. Since a biological brain’s RIs may not be constant and vary with time and individual differences, accurate compensation requires implanting fluorescent beads in every trial, which is a time-consuming and invasive surgical procedure with many steps to obtain a single image. In contrast, our system is less invasive, and neither a high calculation cost nor additional optical devices are necessary. Additionally, there are no critical assumptions about the tissue’s RI.

Moreover, we implemented the Peak-F algorithm for two-photon imaging of living mouse brains. The brains of old mice showed higher RIs than those of young mice at 300–400 μm. However, sensitive to noise, the Peak-C algorithm had difficulty determining the optimal correction collar as old brain tissue tends to show more scattering in deeper areas. Hence, the noise-robust Peak-F algorithm may be helpful for high-resolution observations of biological tissues and for studying the brain’s physical properties. Furthermore, since the spherical aberrant is the mismatching of RIs between water and tissue, the Peak-F algorithm can estimate time-series changes in RIs. In the future, our system may be applicable to indirectly visualize the water dynamics in living biological tissues because the composition of water and lipid in biological tissues determines RIs.

**Fig. 7.**
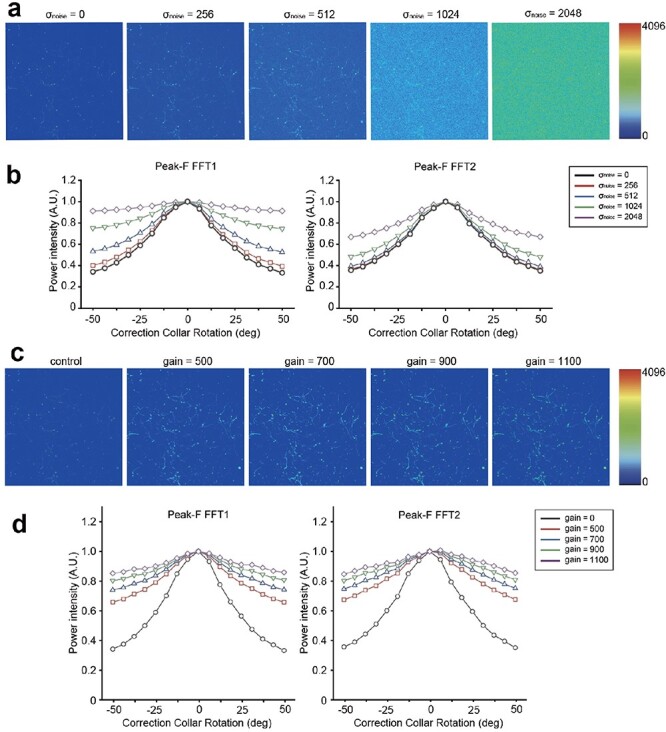
The comparison of Peak-F algorithm (Peak-F FFT1) and that with 2D FFT (Peak-F FFT2) performance on noise-added and saturation-added images. (a) Benchmark image (σ = 0) and that with noise added (σ = 256, 512, 1024 and 2048). (b) Intensities for each of the 17 correction collar positions calculated with the Peak-F FFT1 and Peak-F FFT2 algorithms for the image in a. The vertical axis is the power or contrast intensity, the horizontal axis is the rotation angle of the correction collar and the intensity is normalized by the maximum value. (c) Benchmark image (control) and that with four levels of saturation (gain = 500, 700, 900 and 1100). (d) The intensity of each of the 17 correction collar positions calculated by the Peak-F FFT1 and Peak-F FFT2 algorithms when saturation was applied to the benchmark image in c. The vertical axis is the power or contrast intensity, the horizontal axis is the rotation angle of the correction collar and the intensity is normalized by the maximum value of the control image.

Finally, Peak-F was developed in two variants. The first, Peak-F FFT1, investigated in this study, is a 1D FFT-based spatial frequency algorithm that sums the 2–40 Hz power spectrum of each line in the image (Fig. 3). The second variant, Peak-F FFT2, employs a 2D FFT to calculate the entire image’s 2–40 Hz power spectrum (see Methods). In Fig. 7, we compared the performance of Peak-F FFT1 and FFT2 algorithms concerning their robustness against noise and saturation. While both algorithms demonstrated resilience to noise and saturation in general, FFT2 exhibited superior robustness, especially against noise. Although we implemented FFT1 in the two-photon microscope for this study, future incorporation of FFT2 is anticipated to facilitate a more accurate determination of the optimal correction collar position during *in vivo* imaging.
